# The Undoing of a Diagnosis - Underreporting of Dementia Among Older Adults

**DOI:** 10.1093/geroni/igaf122.4250

**Published:** 2025-12-31

**Authors:** Yuting Qian, Xi Chen

**Affiliations:** Yale School of Public Health, New Haven, Connecticut, United States; Yale University, New Haven, Connecticut, United States

## Abstract

Accurate recognition of dementia is critical for clinical management, care planning, and decision-making. However, stigma, limited awareness, and inadequate disclosure may hinder recognition, and little evidence exists on the extent to which individuals with a documented diagnosis acknowledge it. We evaluated dementia under-reporting, associated factors, and consequences. We used 1998–2020 Health and Retirement Study (HRS)-Medicare linked data of adults aged ≥65 years, weighted for national estimates. Dementia under-reporting was defined as individuals with a dementia diagnosis in claims who did not report dementia in the matching HRS interview. Generalized Estimating Equation (GEE) models with a logit link estimated adjusted under-reporting rates and assessed patient- and provider-level factors, as well as subsequent health care utilization and financial planning. Among 6,134 persons with probable dementia (PWPD), 41% with a dementia diagnosis did not self-report dementia. Under-reporting was highest among self-respondents (66%) compared with 52% for depression, 39% for diabetes, 23% for hypertension, and 17% for arthritis. Rates exceeded 80% before probable symptom onset and remained about 40% afterward. Under-reporting varied significantly by education, living arrangement, dual eligibility, plan type, and diagnosis setting. PWPD who under-reported were less likely to have problem-based visits (OR, 0.71; 95% CI, 0.54-0.95), influenza vaccination (OR, 0.68; 95% CI, 0.55-0.84), and a witnessed will or trust (OR, 0.62; 95% CI, 0.47-0.81). This is the first national longitudinal study to demonstrate the existence and consequences of dementia under-reporting. Beyond improving detection, policies should ensure that diagnosed individuals, especially in preclinical or prodromal stages, are adequately informed.

